# Intra- and inter-fraction breath-hold variations and margins for radiotherapy of abdominal targets

**DOI:** 10.1016/j.phro.2023.100509

**Published:** 2023-11-11

**Authors:** Stefanie Ehrbar, Markus Schrader, Giulia Marvaso, Sophie Perryck, Janita E. Van Timmeren, Matea Pavic, Amanda Moreira, Stephanie Tanadini-Lang, Matthias Guckenberger, Nicolaus Andratschke, Helena Garcia Schüler

**Affiliations:** a Department of Radiation Oncology, University Hospital Zurich, University of Zurich, Zurich, Switzerland; b Department of Radiation Oncology, European Institute of Oncology IRCSS, Milan, Italy; c Department of Oncology and Hemato-oncology, University of Milan, Milan, Italy

**Keywords:** Expiration breath-hold, Abdomen, Margin calculation, Stereotactic body radiotherapy

## Abstract

Radiotherapy in expiration breath-hold (EBH) has the potential to reduce treatment volumes of abdominal targets compared to an internal target volume concept in free-breathing. The reproducibility of EBH and required safety margins were investigated to quantify this volumetric benefit. Pre- and post-treatment diaphragm position difference and the positioning variability were determined on computed tomography. Systematic and random errors for EBH position reproducibility and positioning variability were calculated, resulting in margins of 7 to 12 mm depending on the prescription isodose and fractionation. A reduced volume was shown for EBH for lesions with superior-inferior breathing motion above 4 to 8 mm.

## 1 Introduction

Metastatic liver cancer remains one of the most difficult lesions to treat with stereotactic body radiation therapy (SBRT). SBRT offers an alternative to conventional radiotherapy for patients with unresectable liver metastases with an increasing probability of tumor control and reduced dose to the surrounding normal tissue [1], [2]. In the last years, guidelines and recommendations from large SBRT groups have emerged [3], [4], [5].

Motion management has been a central issue in the development of SBRT over the last decades to improve the tolerability and feasibility of high-dose regimens [6], [7], [8], [9], [10]. The most frequently practiced approach to take the mobility of the target into account is the generation of an internal target volume (ITV) [11], [12]. Breath-hold as a form of gating, either inhalation or exhalation, seems to be a valid option to reduce positional uncertainty of abdominal tumors [13], [14], [15], [16], [17], [18], and might thereby reduce the respiratory safety margins. For reliable gating, target motion tracking is needed. Most commonly used are gating systems based on external signals. However, their correlation to the internal target motion might change [19], [20], [21]. This reproducibility needs to be considered in treatment planning.

A study for pancreatic cancer found that the dose to organs at risk was higher in inhale than in exhale gating position [22]. A recent work compared intra- and interfraction motion on targets in the liver, suggesting a more reproducible situation with exhale breath-hold than inspiration breath-hold and fewer errors [23]. The present study aimed to evaluate the feasibility of an expiration breath-hold (EBH) technique with an external surrogate for SBRT of abdominal tumors concerning target localization reproducibility and subsequent safety margins, to compare treatment volumes between EBH and ITV concepts in free-breathing.

## 2 Materials and methods

### 2.1 Patients and target volumes

From 2017 to 2020, 40 patients (49 treatment plans) were identified as eligible for EBH abdominal SBRT. Patient screening and drop-outs are described in Supplementary Material A. All dropouts occurred at the time of simulation computed tomography (CT). All remaining 21 patients (25 target volumes) completed treatment in EBH and were used for this analysis. Target volumes were located in the liver (21), adrenal gland (3), and pancreas (1). This analysis was approved by the cantonal ethics committee Zurich.

### 2.2 Radiotherapy planning, delivery, and EBH technique

Simulation CT scans were performed, if possible, with intravenous contrast-enhancing (CE) agent, on a Somatom Definition AS CT scanner (Siemens Healthineers). During CT acquisition, the external breathing curve was recorded with the RGSC Respiratory Gating system (Varian Medical Systems), and a gating window of ± 2 mm was set around the EBH position. RGSC uses an externally placed marker block providing a 1D respiration signal. Gross target volume (GTV) delineation was performed on CT in EBH.

The planning target volume (PTV) was defined as GTV plus 10 mm [1]. Treatments were prepared for a Truebeam linear accelerator (Varian Medical Systems), with the highest possible dose rate (flattening filter free, 10 MV, 2400 MU/s). Volumetric modulated arc therapy was used with 2–4 partial arcs (median 3). Single fraction dose varied from 2 Gy to 12.5 Gy (median 7 Gy), in 3 to 28 fractions (median 5). The 25 treatment courses consisted of 154 delivered fractions. Most plans (n = 20) were prescribed on 65% isodose line, others on 80% (n = 4) or homogeneous dose (n = 1) (see Supplementary Table B.1 for patient characteristics).

During treatment, the RGSC gating system was fixed to the patient’s abdomen and used to guide the breath-hold position and interrupt the beam if the position was outside the gating window. Patients were guided with the audio-visual feedback system [24], [25]. Before treatment, gated half-rotation cone-beam CT (CBCT) was performed in EBH. Each setup scan was matched against the simulation CT scan with a bone match to the spine, followed by a soft tissue match to the liver, or GTV if visible. After the first treatment fraction, an additional gated CBCT in EBH was performed in most patients (n = 22, see Supplementary Table B.1).

These simulation CTs and the pre- and post-treatment CBCTs were used to evaluate the inter- and intrafraction breath-hold reproducibility of EBH and the inter-rater positioning variability to determine adequate PTV margins for EBH treatments, as described below.

### 2.3 Inter- and intrafraction reproducibility of breath-hold position

The setup CBCT scans in EBH were used to evaluate the day-to-day (interfraction) reproducibility of EBH. The pre-treatment CBCT of all patients and fractions (154 scans from 25 patient treatment courses) were evaluated. Each setup scan was matched to the simulation CT scan with a bone match to the spine. Then, the difference in the diaphragm position between simulation and treatment was measured as a value for the reproducibility of the breath-hold position. The measurement was taken at the highest point of the diaphragm in the superior-inferior (SI) direction.

For intrafraction reproducibility, all available CBCT scans taken post-treatment were compared to the pre-treatment setup scans (47 scans from 22 patient treatment courses). These scans were likewise matched to the spine and the difference in diaphragm position was measured.

For each patient, the mean and standard deviation of the inter- and intrafraction reproducibility was determined. These were used to calculate the mean, systematic, and random error. The mean error is the average of all patients‘ means, the systematic error is the standard deviation of the patients‘ means, and the random error is the root-mean-square error of the patients‘ standard deviations. The calculation of the errors for the intrafraction reproducibility was based on the patients with more than one post-CBCT scan (36 scans from 10 patient treatment courses, median: 3 per course, see Supplementary Table B.1). The mean inter- and intrafraction diaphragm offsets were tested for significance (p < 0.05) with a Wilcoxon signed rank test.

### 2.4 Inter-rater positioning variability

Since the patients are set up according to the liver position (or GTV if visible) and not the bony anatomy of the daily CBCT scan, the interfraction reproducibility of the EBH position can be omitted in a margin calculation. However, the accuracy of the patient positioning based on soft-tissue matching should be considered instead. For this, three trained observers repeated the target matching of the first CBCT against the simulation CT of each patient. These observer-derived positioning values were compared to the positioning values on the treatment day. The mean, systematic, and random errors were determined as described above.

### 2.5 Margin calculation

For a margin calculation according to van Herk et al. [26], [27] and Gordon et al. [28], all systematic and random errors need to be determined. With the derived errors for the intrafraction breath-hold reproducibility and the inter-rater positioning variability, the required safety margin (*M*) for EBH treatment in the SI direction can be calculated. The margin is given by the systematic errors for tumor delineation (Σ_*delineation*_), tumor positioning (Σ_*positioning*_), and intrafraction breath-hold reproducibility (Σ_*intrafraction*_), and the random errors for tumor positioning (*σ*_*positioning*_), intrafraction breath-hold reproducibility (σ_intrafraction_) and the treatment machine accuracy (*σ*_*machine*_):

(1) $$M=\alpha \sqrt{{\Sigma_{\mathrm{total}}}^{2}-\frac{\sigma_{\mathrm{total}}^{2}}{N_{\mathrm{obs}}}+\frac{\sigma_{\mathrm{total}}^{2}}{N_{\mathrm{treat}}}}+\beta_{\mathrm{heterogeneous}}\sqrt{\frac{\sigma_{\mathrm{total}}^{2}N_{\mathrm{obs}}\left(N_{\mathrm{treat}}-1\right)}{N_{\mathrm{treat}}\left(N_{\mathrm{obs}}-1\right)}}$$

with

(2) $$\Sigma_{\mathrm{total}}=\sqrt{\Sigma_{\mathrm{delineation}}^{2}+\Sigma_{\mathrm{positioning}}^{2}+\Sigma_{\mathrm{intrafracion}}^{2}}$$

and

(3) $$\sigma_{\mathrm{total}}=\sqrt{\sigma_{\mathrm{positioning}}^{2}+\sigma_{\mathrm{intrafraction}}^{2}+\sigma_{\mathrm{machine}}^{2}}$$

Where *N_obs_* is the number of fractions in the error assessment and *N_treat_* is the number of fractions in the treatment for which the margins are calculated. Since the intrafraction EBH reproducibility errors were based on a median of 3 fractions per patient, *N_obs_* = 3 was used. Values for $\beta_{\mathrm{heterogeneous}}$ were assessed in a previous study [29] and α is the value to reach a certain population coverage. Margins were calculated for *N_treat_* = 3,5,6,8 and 10. The values used for the calculation are listed in Table 1.

**Table 1:** Variables used for margin calculation, and calculated margins and threshold motion amplitudes for different fractionations.

	Variable	Value	Comment	
Variables for margin calculation	Σ_*delineation*_	1.0 mm	Half the CT slice thickness	
	Σ_*positioning*_	1.5 mm	See Fig. 1	
	Σ_*intrafraction*_	2.9 mm	See Fig. 1	
	Σ_*positioning*_	1.6 mm	See Fig. 1	
	*σ*_*intrafraction*_	3.9 mm	See Fig. 1	
	*σ*_*machine*_	0.6 mm	90th percentile of the maximum measured radius during routinely performed Winston-Lutz tests [29]	
	*α*	2.5	90% of population, 3D [26], [27]	
	*β*_*heterogeneous*_	0.31/0.85	For 65%/80% isodose prescription [29]	
	*N*_*obs*_	3	Number of fractions in treatments from which errors were derived	

Prescription	Fractions (*N*_*treat*_)	Margin EBH SI (*M*_*SI,EBH*_)	Margin ITV (*M*_*ITV*_)	Threshold motion amplitude (*h_th_*)

65% isodose	3	9.9 mm	5.0 mm	6.5 mm
	5	8.5 mm	4.7 mm	5.1 mm
	6	8.2 mm	4.6 mm	4.8 mm
	8	7.6 mm	4.3 mm	4.4 mm
	10	7.3 mm	4.1 mm	4.3 mm

80% isodose	3	12.2 mm	6.0 mm	8.3 mm
	5	11.1 mm	5.7 mm	7.2 mm
	6	10.7 mm	5.6 mm	6.8 mm
	8	10.3 mm	5.4 mm	6.5 mm
	10	10.0 mm	5.2 mm	6.4 mm

### 2.6 Target volume comparison

Theoretical PTV volumes (*PTV_EBH_* and *PTV_ITV_*) based on a circular GTV with different radii (*r*) and for different motion amplitudes (*h*) were derived. The different PTV margins for the EBH concept (*M_SI__,__EBH_*, *M_AP__,__EBH_*, and *M_LR__,__EBH_* for SI, anterior-posterior (AP), and left–right (LR) motion, respectively), and the PTV margin for the ITV concept (*M_ITV_*) were calculated according to the formula 1 with *N_treat_* = 5 and 65%-isodose prescription. For *M_ITV_*, the intrafraction errors were set to zero in formulas 2 and 3. The *GTV*, *ITV, PTV_EBH_*, and *PTV_ITV_* volumes are then given by the following formulas:

(4) $$\mathrm{GTV}=\frac{4\pi }{3}*r^{3}$$

(5) $$\mathrm{ITV}=\frac{4\pi }{3}*r^{3}+\pi *h*r^{2}$$

(6) $${\mathrm{PTV}}_{\mathrm{EBH}}=\frac{4\pi }{3}*\left(r+M_{\mathrm{SI},\mathrm{EBH}}\right)*\left(r+M_{\mathrm{AP},\mathrm{EBH}}\right)*\left(r+M_{\mathrm{LR},\mathrm{EBH}}\right)$$

(7) $${\mathrm{PTV}}_{\mathrm{ITV}}=\frac{4\pi }{3}*{\left(r+M_{\mathrm{ITV}}\right)}^{3}+\pi *h*{\left(r+M_{\mathrm{ITV}}\right)}^{2}$$

For this estimation, only motion in the SI direction was assumed. Therefore, the margins *M_AP__,__EBH_* and *M_LR__,__EBH_* were set equal to *M_ITV_.* Using these margins, the threshold motion amplitude (*h_th_*) for equality of *PTV_EBH_* and *PTV_ITV_* can be derived:

(8) $$1=\frac{{\mathrm{PTV}}_{\mathrm{EBH}}}{{\mathrm{PTV}}_{\mathrm{ITV}}}=\frac{\frac{4\pi }{3}*\left(r+M_{\mathrm{SI},\mathrm{EBH}}\right)*\left(r+M_{\mathrm{ITV}}\right)*\left(r+M_{\mathrm{ITV}}\right)}{\frac{4\pi }{3}*{\left(r+M_{\mathrm{ITV}}\right)}^{3}+\pi *h_{\mathrm{th}}*{\left(r+M_{\mathrm{ITV}}\right)}^{2}}=\frac{r+M_{\mathrm{SI},\mathrm{EBH}}}{r+M_{\mathrm{ITV}}+\frac{3}{4}h_{\mathrm{th}}}$$

(9) $$h_{\mathrm{th}}=\frac{4}{3}\left(M_{SI,\mathrm{EBH}}-M_{\mathrm{ITV}}\right)$$

A volumetric benefit for EBH is given for tumors with motion larger than *h_th_*.

## 3 Results

**Fig. 1:**
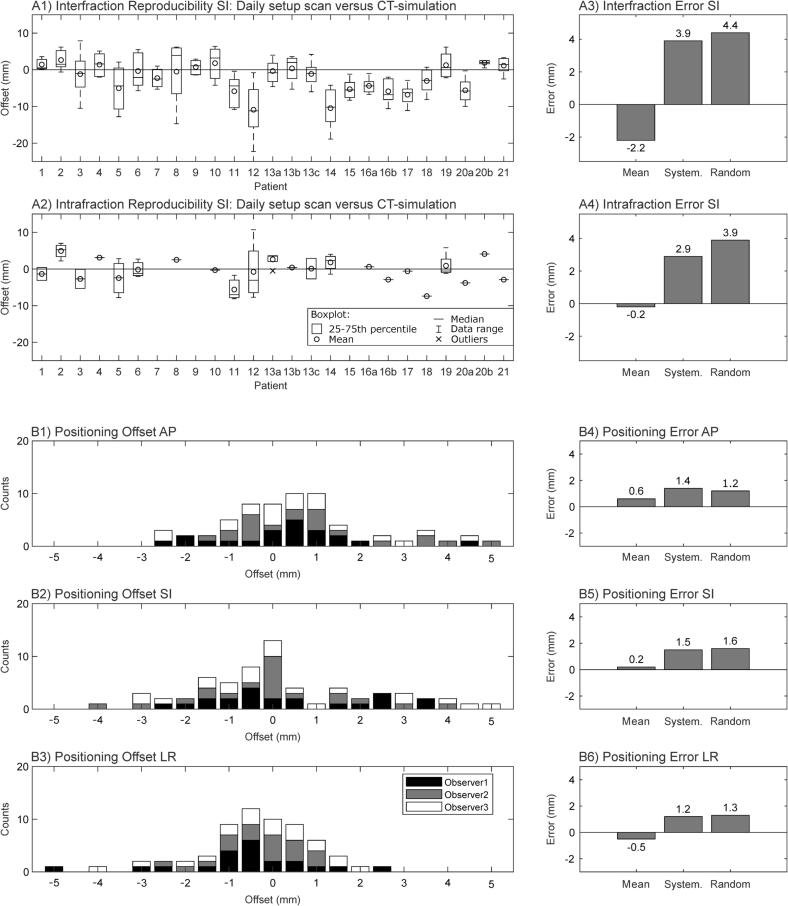
Reproducibility of expiration breath-hold position and inter-rater positioning variability. The difference in diaphragm position (superior-inferior SI) after the match on bony anatomy for inter- (A1) and intrafraction (A2) changes. Mean, systematic, and random errors of the inter- (A3) and intrafraction (A4) breath-hold position. Positive/negative values: The diaphragm is more cranial/caudal than in the reference image. Histograms of the positioning offsets for the three observers versus the online matched values for translation in anterior-posterior (AP, B1), superior-inferior (SI, B2), and left–right (LR, B3). Mean, systematic, and random errors of the inter-rater positioning variability for AP (B4), SI (B5), and LR (B6).

The inter- and intrafraction diaphragm offsets (in SI direction) are shown as boxplots for each patient in Fig. 1, together with the mean, systematic, and random errors of the EBH position. The values were slightly larger for the interfraction reproducibility (mean error: −2.2 mm, systematic: 3.9 mm, random: 4.4 mm) than the intrafraction reproducibility (mean error: −0.2 mm, systematic: 2.9 mm, random: 3.9 mm). The interfraction mean offset showed a statistically significant difference from zero (p = 0.04), resulting in more caudal positioning of the liver before treatment compared to simulation. For the intrafraction mean offset, no significance was found (p > 0.1). The inter-rater positioning variability is also depicted in Fig. 1. For all directions, similar errors were found. The mean, systematic, and random errors for the SI direction were 0.2 mm, 1.5 mm, and 1.6 mm, respectively.

Calculated safety margins for EBH and ITV concepts in the SI direction are listed in Table 1. The required margins for EBH ranged from 7 mm (10 fractions) to 10 mm (3 fractions) in the case of a 65%-isodose prescription. For an 80%-isodose prescription, margins of 10 mm (10 fractions) to 12 mm (3 fractions) were calculated.

A volumetric benefit of EBH over the ITV concept in free breathing was given for threshold motion amplitudes ranging from 4 mm to 8 mm, depending on the prescription (see Table 1).

## 4 Discussion

Safety margins for abdominal SBRT in expiration breath-hold with audio-visual support and external 1D motion surrogate have been derived based on the intrafraction breath-hold reproducibility and the inter-rater positioning variability of patients treated with this technique. Although the derived safety margins for EBH are large with 7 to 12 mm, depending on prescription isodose and number of fractions, a reduction in irradiated volume for EBH compared to the conventional ITV concept was shown for motion amplitudes above 4 to 8 mm.

Interfraction breath-hold variability was slightly larger than intrafraction variability. While interfraction variability can be compensated with daily imaging, the intrafraction breath-hold reproducibility is still the largest contributor to the required margin for EBH with external motion monitoring: Although the position of the external surrogate was stable within ±2 mm during the repeated breath-holds, the internal diaphragm position showed intrafraction variability ranging from −8 to 11 mm.

Oliver et al. [23] used the same external surrogate system to guide the breath-hold and investigated reproducibility errors in deep inspiration breath-hold (DIBH) as well as deep expiration (DEBH). Our errors for normal EBH are larger than their DEBH errors, but similar to their DIBH errors. The use of deep expiration might give additional stability.

On top of volume reduction, EBH radiotherapy has further potential advantages compared to free-breathing treatments: Reduction in imaging motion artifacts allows for easier image fusion and higher image quality of CBCT, and thereby facilitates soft-tissue matching showing low variations among the therapists with inter-rater positioning errors below 2 mm.

The limitations of our analysis are the retrospective character, the relatively small patient cohort, and the assumption of only superior-inferior motion for the margin calculation and volumetric analysis. Also, this evaluation is based on pre- and post-treatment images and might not correctly depict the position variation during treatment. As only an external surrogate was used for control of the breath-hold level, treatment volumes might be further reduced with internal motion monitoring. Additionally we assumed that the patient (bony anatomy) did not move during the treatment. This limitation of our study was because pre- and post-treatment images were not in the same frame of reference.

Based on our patient cohort, we were able to derive adequate safety margins for abdominal SBRT in EBH and to define respiratory motion amplitude thresholds to identify patients who might benefit from treatment in EBH.

## CRediT authorship contribution statement

**Stefanie Ehrbar:** Investigation, Formal analysis, Methodology, Writing – original draft. **Markus Schrader:** Writing – original draft. **Giulia Marvaso:** Investigation, Data curation. **Sophie Perryck:** Project administration, Writing – review & editing. **Janita E. Van Timmeren:** Formal analysis, Methodology. **Matea Pavic:** Resources, Writing – review & editing. **Amanda Moreira:** Investigation, Data curation. **Stephanie Tanadini-Lang:** Project administration, Supervision, Methodology, Conceptualization. **Matthias Guckenberger:** Resources, Writing – review & editing. **Nicolaus Andratschke:** Resources, Writing – review & editing. **Helena Garcia Schüler:** Resources, Supervision, Project administration, Writing – review & editing, Conceptualization.

## Declaration of Competing Interest

The authors declare that they have no known competing financial interests or personal relationships that could have appeared to influence the work reported in this paper.
