# Supplementary material for: Intra- and inter-fraction breath-hold variations and margins for radiotherapy of abdominal targets
Source: Phys Imaging Radiat Oncol. 2023 Nov 11;28:100509. doi: 10.1016/j.phro.2023.100509 (PMC10692905; doi:10.1016/j.phro.2023.100509)
Supplement: Supplementary Data 1 [file mmc1.pdf]

Supplementary Material

Intra- and inter-fraction breath-hold variations and margins for radiotherapy of abdominal targets

A. Screening for treatment in expiration breath-hold

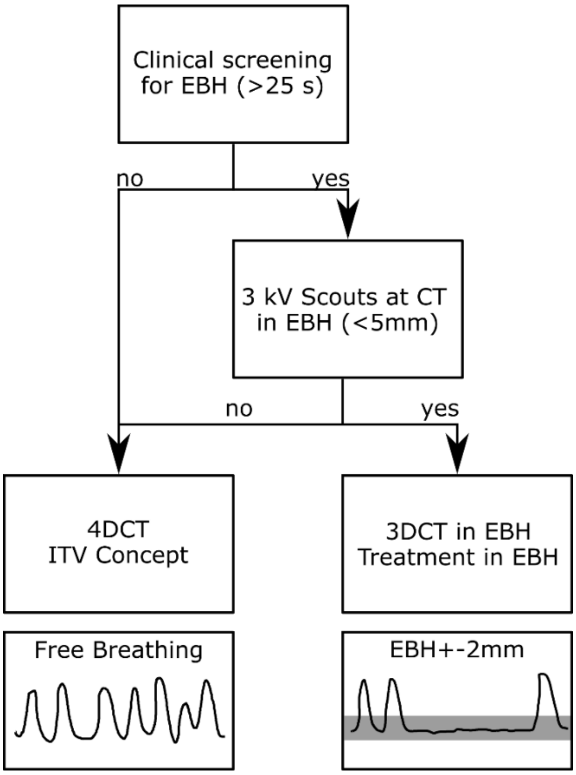

Figure A.1: Screening workflow for treatment in expiration breath-hold (EBH).

Patients were instructed to train to hold their breath in expiration at home. Prior to simulation computed tomography (CT), they were tested for EBH duration (>25 s). The patients performed a couple of dry runs with the audio-visual feedback system while lying on the CT table. If successful, the internal reproducibility of the diaphragm in EBH is examined with 3 repeated kV scouts (<5 mm). If successful, the patient is scanned and treated in EBH. Else, the patient is scanned in free-breathing and treated with the conventional internal target volume (ITV) concept.

From February 2017 to March 2020, 40 patients (49 treatment plans) were identified as eligible for EBH abdominal SBRT. Patient exclusion criteria before and during the study were the following: insufficient breath-hold duration (<25 s) during computed tomography (CT) scan (n=13), treatment cancellation (n=2), intolerance to prolonged table time (n=1) and a relevant anatomical shift (n=1). In two patients no target movement was observed, thus no motion compensation was needed. All remaining 21 patients (25 target volumes) showed less than 5 mm variation in diaphragm position on three repeated CT scouts, completed treatment in EBH and were used for analysis.

B. Patient cohort

Table B.1: Patient characteristics.

Patient	# Fractions	Total Dose (Gy)	Location	GTV (cc)	# post-treatment CBCT
1	3	36	Liver	126.9	2
2	5	35	Liver	9.7	4
3	28	56	Liver	13.7	2
4	5	20	Pancreas	3.3	1
5	3	37.5	Liver	37.4	3
6	5	30	Liver	39.8	3
7	3	30	Adrenal gland (right)	6.3	0
8	5	30	Liver	69.4	1
9	5	35	Liver	128.2	0
10	3	37.5	Liver	2.3	1
11	5	50	Liver	1.0	3
12	5	45	Liver	30.1	4
13a	10	50	Liver	0.3	5
13b	5	40	Liver	0.4	1
13c	5	40	Liver	0.5	2
14	10	50	Liver	62.8	4
15	5	50	Liver	5.7	0
16a	5	35	Adrenal gland (right)	68.4	1
16b	5	35	Adrenal gland (left)	3.9	1
17	8 (10)*	24 (30)*	Liver	19.5	1
18	5	45	Liver	7.7	1
19	6	30	Liver	6.8	4
20a	5	40	Liver	0.8	1
20b	5	40	Liver	1.1	1
21	5	25	Liver	201.3	1

** Patient did not complete treatment. a, b, c: different lesions*
GTV: Gross tumor volume, CBCT: cone-beam computed tomography
